# ITRAQ Proteomic Analysis of Yellow and Black Skin in Jinbian Carp (*Cyprinus carpio*)

**DOI:** 10.3390/life10100226

**Published:** 2020-09-30

**Authors:** Xiangchen Ye, Lingling Zhou, Jingyi Jia, Lingjing Wei, Yanhong Wen, Xueyu Yan, Jie Huang, Baojiang Gan, Kang Liu, Yejian Lv, Guangfu Hu

**Affiliations:** 1 Aquatic Species Introduction and Breeding Center of Guangxi, Nanning 530031, China; chen-chen79@163.com (X.Y.); ljwei9012@163.com (L.W.); yanxueyu@bbgu.edu.cn (X.Y.); ganbaojiangvip@126.com (B.G.); liukande1988@163.com (K.L.); 2 College of Fisheries, Huazhong Agricultural University, Wuhan 430070, China; llz9872@163.com (L.Z.); Jiajy94@163.com (J.J.); 3 Extension Station of Fisheries Technology of Liuzhou, Liuzhou 545006, China; Wenyanhong888@163.com (Y.W.); huangjiexd@126.com (J.H.)

**Keywords:** pigmentation, ITRAQ, Jinbian carp, melanin, pteridine

## Abstract

Colors are important phenotypic traits for fitness under natural conditions in vertebrates. Previous studies have reported several functional genes and genetic variations of pigmentation, but the formation mechanisms of various skin coloration remained ambiguous in fish. Jinbian carp, a common carp variant, displays two colors (yellow and black) in the skin, thus, it is a good model for investigating the genetic basis of pigmentation. In the present study, using the Jinbian carp as model, isobaric tags for relative and absolute quantification (ITRAQ) proteomics analysis was performed for yellow and black skin, respectively. The results showed that 467 differentially expressed proteins (DEPs) were identified between the yellow skin and the black skin. Similar to mammals, the up-regulated DEPs in black skin included UV excision repair protein RAD23 homolog A (Rad23a), melanoregulin (mreg), 5,6-dihydroxyindole-2-carboxylic acid oxidase5 (tyrp1) and melanocyte protein PMEL (PMEL), which were mainly grouped into melanogenesis pathway. However, several up-regulated DEPs in yellow skin were mainly enriched in nucleotide metabolism, such as GTPase IMAP family member 5 (GIMAP5), AMP deaminase 1 (AMPD1), adenosylhomocysteinase b (ahcy-b), and pyruvate kinase (PKM). In summary, several candidate proteins and their enrichment pathways for color variation in Jinbian carp were identified, which may be responsible for the formation of different colorations.

## 1. Introduction

Colors are important phenotypic traits for fitness under natural conditions in vertebrates [1]. Skin coloration is the result of diverse pigments synthesized by chromatophores, which is affected by several factors, including environmental, nutritional, physiological, and genetic conditions. Among them, the genetic basis of skin pigmentation is the most fundamental and important factor. Therefore, several studies have been devoted to examine the genetic basis of pigmentation in animals. Previous studies have found that many biological pathways and genes were involved in the pigment synthesis, including melanin and pteridine synthesis pathway [2].

In teleost, several chromatophores have been identified, which played an important role in the formation of variety coloration, including melanophores (melanin granules), xanthophores (pteridine or carotenoid granules), iridophores (guanine), leucophores, and erythrophores (carotenoids and pteridine) [3,4,5,6]. Using teleost as models, several studies have tried to examine the genetic variation among different colored skin. Haffter et al. observed that dominant mutations in genes could change the pigment pattern in adult zebrafish [7]. However, the key genes and biological processes, which are involved in the combination of different colors, are still ambiguous.

Common carp (*Cyprinus carpio L.*) was the most widely cultivated freshwater fish in the world. In China, common carp has been farmed for more than 2500 years [8,9]. During the long farming history, common carp has evolved several variants, which was caused by geographic isolation and natural as well as human selection pressures [10]. In China, several variants have also been developed in the regional distribution and cultivation zones over thousands of years. Among them, Jinbian carp is an important variant for paddy-field fish culture in China, which appears with two yellow stripes on both sides of its dorsal fin [11]. Due to its variable colors, Jinbian carp is a good system for studying color pattern polymorphism.

In this study, isobaric tags for relative and absolute quantification (ITRAQ) analysis was used to examine the proteomic variations between yellow skin and black skin in Jinbian carp. The aims of our present study were to: (i) provide an overview of the proteome in yellow skin and black skin; (ii) identify differentially expressed proteins (DEPs) that were possibly involved in yellow coloration; (iii) examine the expression levels of key proteins in the melanin and pteridine pathways between two skin colors.

## 2. Materials and Methods

### 2.1. Sample Preparation

**Figure 1:**
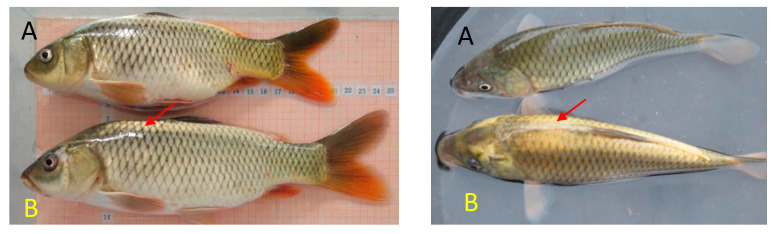
Photographs of the wild common carp (**A**) and Jinbian carp (**B**).

The common carp (with black skin only) and the manually selected Jinbian common carp (with yellow and black skin) (Figure 1) were collected from Aquatic Species Introduction and Breeding Center of Guangxi, Nanning, China. The yellow skin (Y) and black skin (B) were collected from the same Jinbian carp. In addition, the black skin in the wild common carp (W) was also collected. All the samples in the cryogenic vials were immediately frozen in liquid nitrogen and stored at −80 °C until further processing. All animal experiments were conducted in accordance with the guidelines and approval of the respective Animal research and Ethics committees of Huazhong Agricultural University (Ethical Approval No. HBAC20091138; Date: 15 November 2009).

### 2.2. Protein Preparation and iTRAQ Labeling

The skin samples were put into the lysis buffer (8 M urea, 0.3% SDS) with protease inhibitors (Thermo, Rockford, IL, USA), and were then sonicated in ice. The protein samples were reduced with DTT (10 mM) at 56 °C for 1 h, then the IAM (55 mM) were used to alkylate the samples in the darkroom for 1 h. The reduced and alkylated protein mixtures were precipitated by adding 4× volume of chilled acetone at −20 °C overnight. After centrifugation at 4 °C with 30,000× *g*, the pellet was dissolved in 0.5 M TEAB (Applied Biosystems, Milan, Italy) and sonicated in ice. After centrifugation at 4 °C with 30,000× *g*, an aliquot of the supernatant was taken to determine the protein concentration with a 2-D Quant Kit (GE Healthcare). The proteins in the supernatant were kept at −80 °C for further analysis. The proteins were digested with modified trypsin at 37 °C overnight and then labeled with different iTRAQ reagents according to the manufacturer’s instruction. The labeled reagent was dissolved in acetonitrile, mixed with the peptides, and then incubated at room temperature for 2 h. The labeled samples were then mixed, desalted, and vacuum-dried. More details for iTRAQ labeling were provided in our previous studies [12].

### 2.3. Liquid Chromatography Tandem Mass Spectrometry (LC/LC-MS/MS) Analysis

Each fraction was resuspended with loading buffer (5 mM ammonium formate containing 2% acetonitrile; pH = 10) and separated by high-pH reversed-phase liquid chromatography (RPLC, Acquity Ultra Performance LC; Waters, Milford, MA, USA). The solvent A and solvent B was 2% ACN (pH = 10, adjusted by ammonia) and 80% ACN (pH = 10, adjusted by ammonia), respectively. The gradient elution was performed with 0–30% solvent B for 2–38 min and 30–100% solvent B for 38–40 min on a high-pH RPLC column (C18, 1.7 µm, 2.1 mm × 150 mm; Waters Corporation, Milford, MA, USA). All mass spectrometry proteomics data were deposited in Integrated Proteome Resources (iProX, http://www.iprox.org/; project ID: IPX0002447000; ProteomeXchange ID: PXD021293).

### 2.4. Analysis of the Identified Proteins

By using Proteome Discoverer^TM^ Software 2.1 (Thermo, Rockford, IL, USA), the raw data obtained from LC/LC−MS/MS analysis were processed. The proteins were annotated with the Blast2GO (Gene Ontology) program against the nonredundant protein database (NR; NCBI) (https://www.blast2go.com/). The transcriptome data from black and yellow skin of Jinbian carp as well as black skin of common carp was also used as a reference database for protein annotation. In addition, the differentially expressed proteins (DEPs) were further assigned to the Kyoto Encyclopedia of Genes and Genomes (KEGG) database (KEGG; http://www.genome.jp/kegg/). The hypergeometric distribution was used for the GO and KEGG pathway enrichment analysis, and Fisher’s exact test was used for accurate test, and Benjamini was used for the correction of multiple tests. In this analysis, the threshold of significant enrichment of GO function and KEGG pathway of DEPs was *p*-value < 0.05. The *p*-value of the significant difference between the samples was calculated by using the *t*-test in the R language. For identifying significantly up- or down-regulated proteins (*p*-value < 0.05), the threshold values of Y/B or Y/W ratios were ≥1.50 or ≤0.67 (≥1.5-fold), respectively.

## 3. Results

### 3.1. Protein Identification and Quantitation

**Figure 2:**
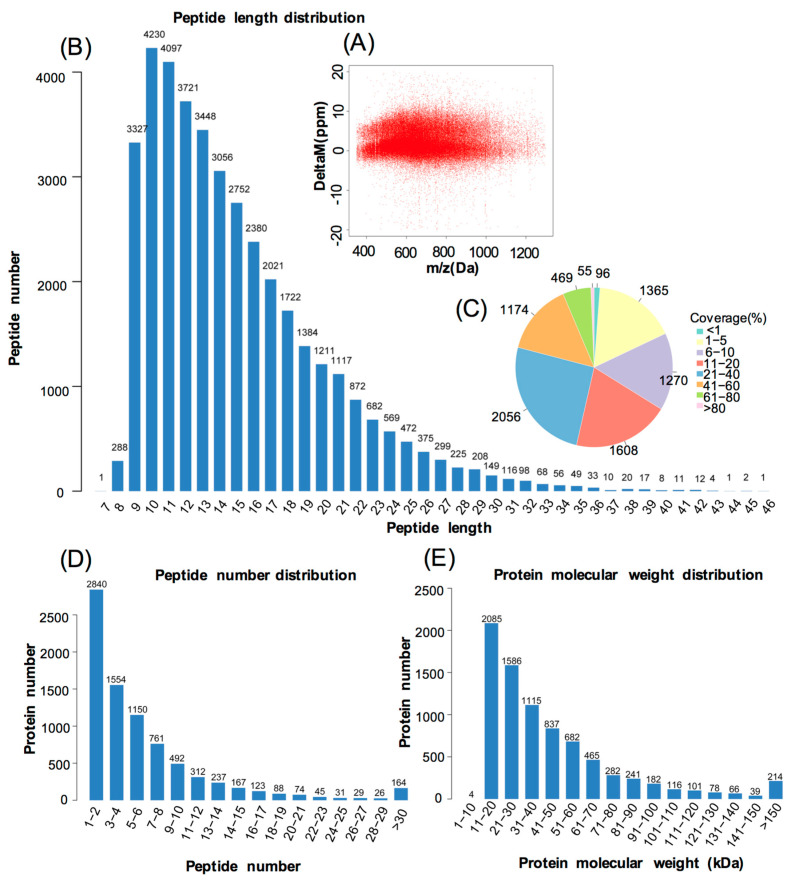
Identification and analysis of the proteome in yellow skin in Jinbian carp (Y), black skin in Jinbian carp (**B**) and black skin in wild common carp (W). (**A**) Distribution of peptide segment matching error. (**B**) The distribution of peptide length. The x-axis indicates the length of the peptide, and the y-axis indicates the number of peptides of the corresponding length. (**C**) The coverage distribution of identified proteins. Each fan represents the proportion of a range of coverage. The larger the fan area, the more proteins covered in this range. The number outside the fan indicates the number of proteins covered in this range. (**D**) The distribution of peptide number. The x-axis indicates the number of peptides covering the protein, and the y-axis indicates the number of proteins. (**E**) The distribution of protein molecular weight. The x-axis shows the size of the identified protein molecular weight (unit: kilodalton, kDa), and the y-axis represents the number of identified proteins reflecting the corresponding size.

The iTRAQ analysis revealed that 8064 protein hits were detected in the common carp skin by Proteome Discover (Supplementary Table S1). The error in the matching of the peptide segment detected in the database was below 0.05 Da (Figure 2A). As shown in Figure 2B, the peptide length was around in 9–30 aa, and most of peptide segments were enriched in 9–16 aa length. The numbers of peptides identified into the proteins was exhibited in Figure 2C, which showed that the coverage of most protein sequence was at 1–40%. In addition, the molecular weight of most proteins was 11–150 kDa (Figure 2E).

### 3.2. Identification and GO Classification of the Proteins in Common Carp Skin

**Figure 3:**
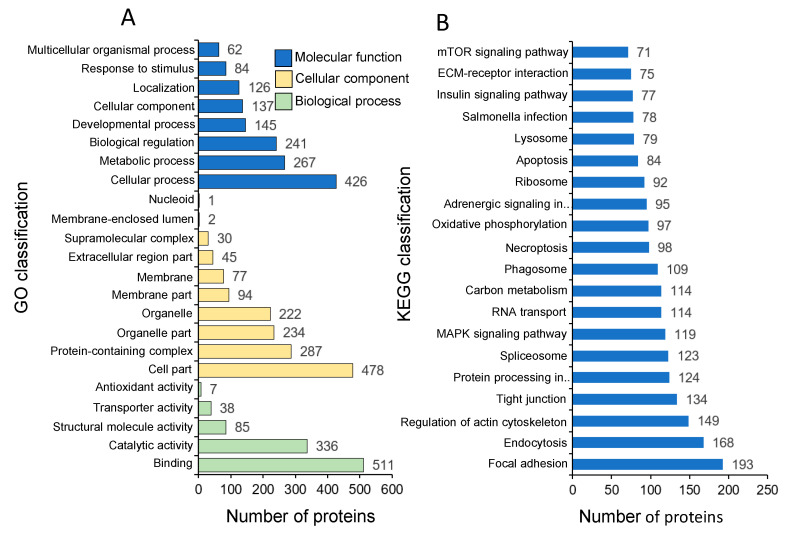
GO (Gene Ontology) (**A**) and KEGG (Kyoto Encyclopedia of Genes and Genomes) (**B**) analyses of the proteins derived from common carp skins.

The annotated proteins were enriched in three GO categories, including biological function, molecular function, and cellular component (Figure 3A). In the molecular functions, most proteins in common carp skins were enriched in the binding (511 proteins), catalytic (336), structural molecule (85), transporter (38), and antioxidant activities (seven). In the cellular component, common carp skin proteins were enriched in the categories of cell part (478 proteins), protein-containing complex (287), organelle part (234), membrane part (94), extracellular region part (45), supramolecular complex (30), and membrane-enclosed lumen (two). The most represented biological functions included the categories of cellular process (426 proteins), metabolic process (267), and biological regulation (241). Other important biological function allocations included the developmental process (145 proteins), cellular component organization (137), localization (126), response to stimulus (84), and multicellular organismal process (62).

Total of 2193 proteins were enriched in 159 pathways. The top 20 pathway ontologies were shown in Figure 3B. Proteins were most enriched in relation to focal adhesion (193 proteins), endocytosis (168), regulation of actin cytoskeleton (149), tight junction (134), protein processing in endoplasmic reticulum (124), spliceosome (123), MAPK signaling pathway (119), RNA transport (114), Carbon metabolism (114), phagosome (109), necroptosis (98), oxidative phosphorylation (97), adrenergic signaling in cardiomyocytes (95), ribosome (92), apoptosis (84), lysosome (79), salmonella infection (78), insulin signaling pathway (77), ECM-receptor interaction (75), and mTOR signaling pathway (71).

### 3.3. Identification of Differentially Expressed Proteins (DEPs) between Yellow and Black Skin in Jinbian Carp

**Figure 4:**
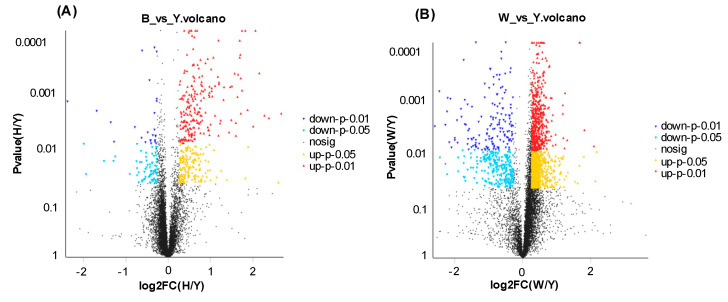
Volcano plot of DEPs in B, Y, and W. (**A**) Volcano plot of DEPs in B and Y. (**B**) Volcano plot of DEPs in W and Y. Splashes represent different genes, Yellow dots indicate proteins that are significantly up-regulated at *p* < 0.05, red dots indicate proteins that are significantly up-regulated at *p* < 0.01, light blue dots indicate proteins that are significantly down-regulated at *p* < 0.05, blue dots indicate protein that are significantly down-regulated under *p* < 0.01 conditions, and black spots are non-significantly differentially expressed proteins.

**Figure 5:**
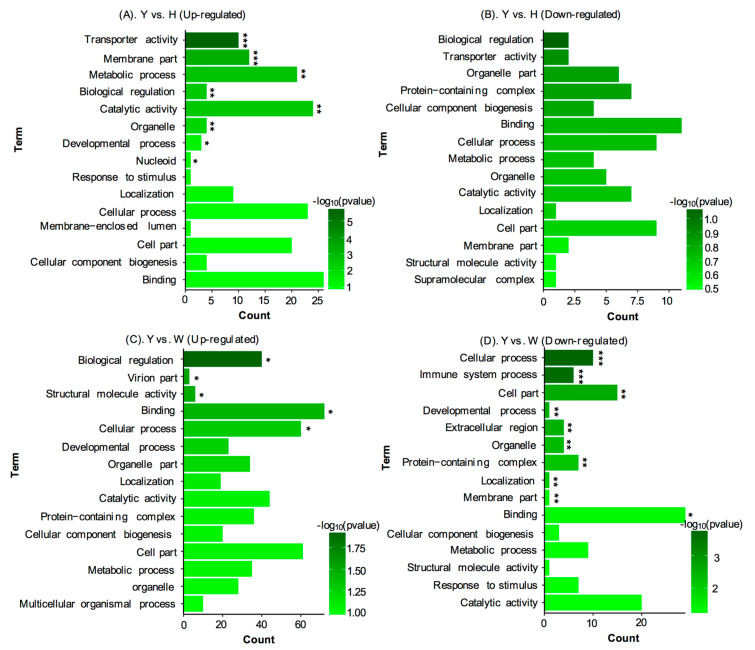
Gene ontology (GO) enrichment analysis of DEPs among Y, B, and W. Statistics of top 15 enriched GO terms for DEPs in yellow skin (**A**) and black skin (**B**) in Jinbian carp; Statistics of top 15 enriched GO terms for DEPs in Y (**C**) and W (**D**); *p* < 0.001 is marked as “***”, *p* < 0.01 is marked as “**”, and *p* < 0.05 is marked as “*”.

A total of 467 DEPs were identified between yellow (Y) and black (B) skin derived from the same Jinbian carp (FC ≥ 1.2 and *p*-value < 0.05) (Figure 4A), including 94 up-regulated proteins (Table 1) and 373 down-regulated proteins (Table 2) in yellow skin. GO analysis showed that most up-regulated DEPs in yellow skin were preferentially involved in metabolic process, transporter activity, catalytic activity, membrane part, biological regulation, organelle, cellular process, and binding (Figure 5A). Additionally, most down-regulated DEPs in yellow skin compared to black skin were mainly grouped into biological regulation, transporter activity, organelle part, protein-containing complex, cellular component biogenesis, binding, cellular process, metabolic process, organelle, catalytic activity, and cell part (Figure 5B).

**Figure 6:**
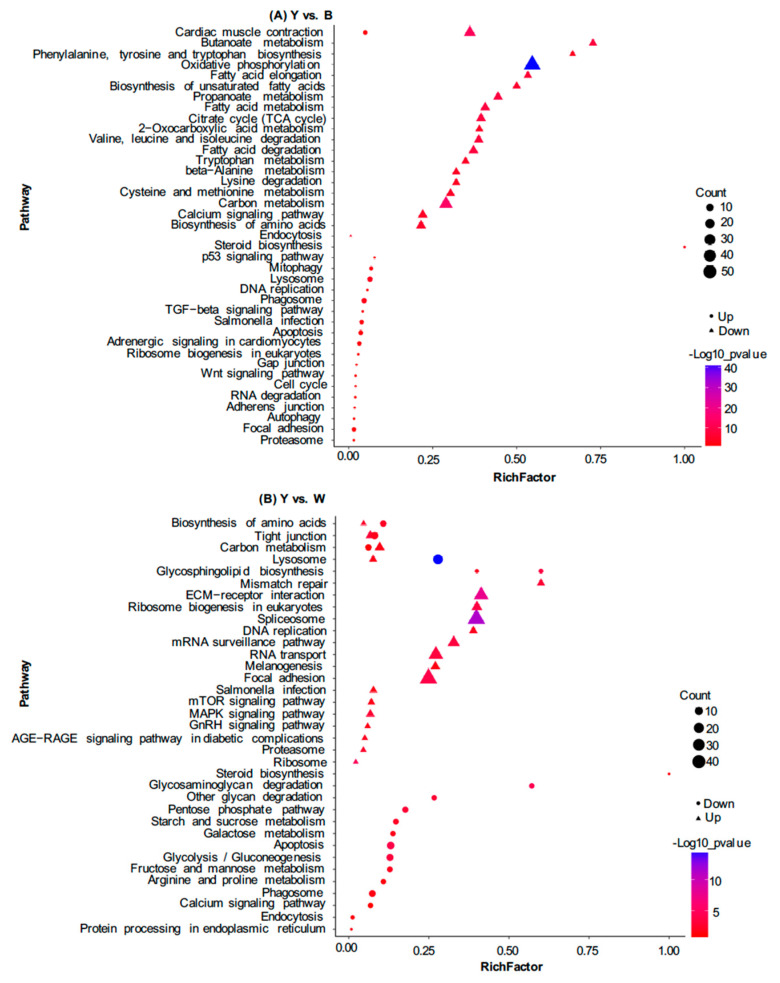
Kyoto Encyclopedia of Genes and Genomes (KEGG) pathway enrichment analysis for DEPs in Y, B, and W. Statistics of top 20 enriched pathways for DEPs in Y and B (**A**); Statistics of top 20 enriched pathways for DEPs in Y and W (**B**). The abscissa represents the enrichment factor.

In addition to the GO analysis, KEGG pathway enrichment analysis was also used to further elucidate the putative functions of these DEPs. These results demonstrated that down-regulated proteins in yellow skin were mainly involved in the oxidative phosphorylation (53/142), carbon metabolism (33/142), cardiac muscle contraction (22/142), biosynthesis of amino acids (14), fatty acid metabolism (13), calcium signaling pathway (13), fatty acid degradation (13), citrate cycle (13), necroptois (12), propanoate metabolism (12), valine, leucine and isoleucine degradation (12), glycolysis/gluconeogenesis (11), adrenergic signaling in cardiomyocytes (11), cysteine and methionine metabolism (10), purine metabolism (two), biosynthesis of unsaturated fatty acids (8), beta-Alanine metabolism (eight), butanoate metabolism (eight), tryptophan metabolism (eight), cellular senescence (eight), lysine degradation (eight), starch and sucrose metabolism (eight), and fatty acid elongation (eight) (Figure 6A). In addition, the up-regulated proteins in yellow skin were enriched in the phagosome (5/21), lysosome (five), focal adhesion (three), salmonella infection (three), adrenergic signaling in cardiomyocytes (three), cardiac muscle contraction (three), apoptosis (three), mitophagy (two), and MAPK signaling pathway (one) (Figure 6A).

### 3.4. DEP Analysis between Yellow Skin in Jinbian Carp (Y) and Black Skin in Wild Common Carp (W)

A total of 129 DEPs were also identified between Y and W (FC ≥ 1.2 and *p*-value < 0.05 (Figure 4B), among which included 58 up-regulated proteins (Table 3) and 71 down-regulated proteins in Y (Table 4). GO analysis showed that most up-regulated genes in Y were grouped into biological regulation, virion part, structural molecule activity, binding, cellular process, developmental process, organelle part, localization, catalytic activity, protein-containing complex, cellular component biogenesis, cell part, metabolic process, organelle, and multicellular organismal process (Figure 5C). In addition, most down-regulated DEPs in Y compared to W were grouped into cellular process, immune system process, cell part, developmental process, extracellular region, organelle, protein-containing complex, localization, membrane part, and binding (Figure 5D).

KEGG analysis showed that up-regulated genes in Y compared to W were preferentially grouped into lysosome (22/84), apoptosis (11/84), tight junction (10), glycolysis/gluconeogenesis (nine), phagosome (eight), biosynthesis of amino acids (seven), carbon metabolism (seven), pentose phosphate pathway (six), focal adhesion (five), regulation of actin cytoskeleton (five), starch and sucrose metabolism (five), fructose and mannose metabolism (five), salmonella infection (four), galactose metabolism (four), glycosaminoglycan degradation (four), adrenergic signaling in cardiomyocytes (four), calcium signaling pathway (four), cardiac muscle contraction (four), arginine and proline metabolism (four), and MAPK signaling pathway (three) (Figure 6B). In addition, the down-regulated genes in Y compared to W were mainly grouped into spliceosome (49/354), focal adhesion (48), RNA transport (31), ECM-receptor interaction (31), regulation of actin cytoskeleton (24), endocytosis (22), mRNA surveillance pathway (19), protein processing in endoplasmic (17), reticulum (15), phagsome (14), ribosome biogenesis in eukaryotes (14), insulin signaling pathway (14), apoptosis (13), necroptoiss (12), adrenergic signaling in cardiomyocytes (11), carbon metabolism (11), melanogenesis (10), and apelin signaling pathway (10) (Figure 6B).

## 4. Discussion

The common carp has been widely farmed in Europe and Asia. In the long-term breeding process, the common carp has evolved hundreds of strains or varieties that display a rich biodiversity and are diverse in skin colors [13]. Due to their diverse skin colors, the common carp was used as a good model to elucidate the skin pigmentation process. Various fish colorations are determined by the density and position of different pigment cells, which is believed to be mainly controlled by genetic factors. As we know, both the melanin and the pteridine synthesis pathways have been found in teleost. The black pigment, melanin, is generated in melanophores through the melanin synthesis pathway [2], and the pteridine synthesis pathway could produce the yellow or reddish pteridine pigments [14]. Now, several genes have been identified in black and yellow pigment synthesis [2], but the detail molecular mechanisms of different pigment synthesis were not well understood. In the present study, to better understand fish skin color genetics, iTRAQ was used to examine the differentially expressed proteins between the melanin synthesis pathway and pteridine synthesis pathway in common carp.

### 4.1. Skin Proteome Analysis

As a first step, 8064 protein kits were detected in common carp skin. GO analysis showed that most proteins were enriched in the immune system process, including endocytosis, phagosome, necroptosis, lysosome, and salmonella infection. These results confirmed that the major function of fish skin is to act as the first barrier of the immune system. It can provide protection against physical damage and assisted with the maintenance of homoeostasis by minimizing exchange between the animal and the environment. The goblet cells in the skin epidermis are responsible for the production of the mucosal layer [15]. The skin mucus is a composite of defensive molecules, including antibodies, together with factors of both the innate and acquired immune system [16]. The mucins are the major macromolecular components of mucus [17]. Mucins are heavily glycosylated proteins, which impart viscoelastic and rheological properties to mucosal layers [18]. In addition, we also found that many proteins are grouped into transporter activity, binding, and membrane-enclosed lumen in common carp skin proteomes. These results indicate that the skin in common carp also plays an important role in transporting gases, ions, nitrogenous waste products, and nutrients [19].

### 4.2. Up-Regulated Proteins in Black Skin Indicated the Molecular Mechanism of the Melanin Synthesis Pathway

Melanin is mainly synthesized by tyrosine within the melanosome [20,21]. In mammals and birds, two types of melanin are produced, the black or brown eumelanin and the lighter pheomelanin, but only eumelanin has been observed in teleost [22]. Eumelanin is synthesized by tyrosine within the melanosome of melanophores. This requires members of the tyrosinase family (TYP, DCT, and TYRPI) and probably Silver (SILV) [23,24,25,26]. Three melanosomal transporters (OCA2, AIMI, and SLC24A5) are crucial for proper melanin synthesis [27]. In the present study, we also found that several up-regulated proteins (Rad23a, mreg, tyrp1, and PMEL) in black skin were grouped into the melanogenesis pathway, including melanoregulin, melanocyte protein, and the UV excision repair protein, which might be responsible for the melanogenesis in the black skin of Jinbian carp. Given that the zebrafish *golded* mutant caused by slc24a5 deficiency is characterized by delayed and reduced development of melanin pigmentation, the up-regulated SLC25A4, SLC25A5, and SLC25A6 in the black skin may also associate with skin color variation [27,28]. However, we did not detect the different expression of TYP, DCT, and SILV between yellow and black skin, which indicated that these proteins might also play a role in the synthesis of yellow pigment. In addition, consistent with our recent transcriptome analysis, we also discovered that ACTC1, MYH6, and MYH7, involved in adrenergic signaling pathway, and ATP2A1, ATP2A2, ATP5F1, ATP5J, and COX5A, involved in oxidative metabolism pathway, were also up-regulated in black skin compared to yellow skin in proteome analysis [29]. Consequently, these genes could be candidate genes for the formation of yellow or black colors in Jinbian carp.

### 4.3. Up-Regulated Proteins in Yellow Skin Indicated the Molecular Mechanism of the Pteridine Synthesis Pathways

The yellow and reddish pteridine pigments are synthesized from GTP through the pteridine synthesis pathway in xanthophores. Three component pathways are involved in pteridine synthesis. Firstly, the GTP are converted into the tetrahydrobiopterin (H4biopterin), which is a cofactor for neurotransmitter synthesis and tyrosinase activity in melanophores. The second component is the regeneration pathway of oxidized H4biopterin. The third pathway shares several steps with the first one and leads to the formation of the yellow pigments, sepiapterin, and its derivatives [2,14]. During these processes, several genes were involved in the yellow pigmentation, such as the GTP cyclohydrolase I (GchI) [14], 6-pyruvoyltetrahydropterin synthase (Pts), sepiapterin reductase (Spr), xanthine oxidase/xanthine dehydrogenase (Xod/Xdh), and protein associated with Myc (PAM) [30]. In the present study, KEGG analysis showed that up-regulated proteins in yellow skin were preferentially grouped into several metabolism process, such as arginine and proline metabolism, pentose phosphate pathway, glycolysis/gluconeogenesis, fructose and mannose metabolism, carbon metabolism, and galactose metabolism. In addition, a few of the up-regulated proteins in yellow skin were also found to be related to nucleotide metabolism, such as GTPase IMAP family member 5 (GIMAP5), AMP deaminase 1 (AMPD1), adenosylhomocysteinase b (ahcy-b), and pyruvate kinase (PKM). These proteins may also play an important role in the yellow pigmentation.

In summary, we conducted a proteomic analysis among the yellow and black skin of Jinbian carp and the black skin of the wild common carp by using iTRAQ technology, and the results indicated that several up-regulated DEPs in black skin, including Rad23a, mreg, tyrp1, and PMEL, and several up-regulated DEPs in yellow skin, including GIMAP5, AMPD1, ahcy-b, and PKM, might be involved in the color variation in Jinbian carp.

## Figures and Tables

**Table 1:** The up-regulated differentially expressed proteins (DEPs) in black skin (B) compared to yellow skin (Y).

Uniprot_ID	Gene	Description	FC(B/Y)	*p* Value
MLRB_CHICK	*N/A*	Myosin regulatory light chain 2B	6.34	0.0029041
MYL4_HUMAN	*MYL4*	Myosin light chain 4	5.22	0.0035055
TNNT2_CHICK	*TNNT2*	Troponin T	4.44	0.0005641
MYPC3_CHICK	*MYBPC3*	Myosin-binding protein C	4.16	7.544 × 10^−5^
TNNT3_HUMAN	*TNNT3*	Troponin T	4.01	0.0049163
TNNI1_HUMAN	*TNNI1*	Troponin I	3.53	2.086 × 10^−5^
MYH7_HUMAN	*MYH7*	Myosin-7	3.53	0.0402934
AT2A1_MAKNI	*atp2a1*	Sarcoplasmic/endoplasmic reticulum calcium ATPase 1	3.28	0.0028990
TPM1_LIZAU	*N/A*	Tropomyosin alpha-1 chain	3.27	0.0002662
TNNI3_XENLA	*tnni3*	Troponin I	3.22	0.0001500
COX42_THUOB	*N/A*	Cytochrome c oxidase subunit 4 isoform 2	3.11	0.0014771
TNNI1_MOUSE	*Tnni1*	Troponin I	3.03	0.0042539
ACTN4_CHICK	*ACTN4*	Alpha-actinin-4	2.82	0.0037840
ACTN2_CHICK	*ACTN2*	Alpha-actinin-2	2.75	0.0009886
HHATL_HUMAN	*HHATL*	Protein-cysteine N-palmitoyltransferase HHAT-like protein	2.71	0.0062292
MYPC1_HUMAN	*MYBPC1*	Myosin-binding protein C	2.61	0.0433731
NNTM_HUMAN	*NNT*	NAD(P) transhydrogenase	2.50	0.0009916
KCRB_CHICK	*CKB*	Creatine kinase B-type	2.48	0.0016669
PYGM_MACFA	*PYGM*	Glycogen phosphorylase	2.36	0.0038506
IDHP_MACFA	*IDH2*	Isocitrate dehydrogenase [NADP]	2.34	0.0043346
AT2A2_CHICK	*ATP2A2*	Sarcoplasmic/endoplasmic reticulum calcium ATPase 2	2.30	0.0008799
ACTC_RAT	*Actc1*	Actin	2.27	0.0071161
AATM_RAT	*Got2*	Aspartate aminotransferase	2.25	0.0006509
MYH7_PIG	*MYH7*	Myosin-7	2.22	0.0386928
UCRI_AOTAZ	*UQCRFS1*	Cytochrome b-c1 complex subunit Rieske	2.15	0.0031625
SRCA_MOUSE	*Srl*	Sarcalumenin	2.09	0.0002984
NEBU_HUMAN	*NEB*	Nebulin	1.96	0.0014726
ECHA_MOUSE	*Hadha*	Trifunctional enzyme subunit alpha	1.96	0.0102789
NDUA4_DANRE	*ndufa4*	Cytochrome c oxidase subunit NDUFA4	1.96	0.0069833
MYH6_MOUSE	*Myh6*	Myosin-6	1.93	0.0204875
MYH7_MOUSE	*Myh7*	Myosin-7	1.91	0.0294791
COX5A_MOUSE	*Cox5a*	Cytochrome c oxidase subunit 5A	1.90	0.0003158
MYH7_HORSE	*MYH7*	Myosin-7	1.84	0.0194304
MDHM_FRAAN	*MMDHI*	Malate dehydrogenase	1.82	0.0010926
GLYM_RABIT	*SHMT2*	Serine hydroxymethyltransferase	1.82	0.0453498
DESM_PIG	*DES*	Desmin	1.79	0.0065272
ATPA_PIG	*ATP5F1A*	ATP synthase subunit alpha	1.78	0.0011789
ATP5J_PONAB	*ATP5J*	ATP synthase-coupling factor 6	1.77	0.0148907
ACSL1_CAVPO	*ACSL1*	Long-chain-fatty-acid--CoA ligase 1	1.75	0.0038413
MYO6_CHICK	*MYO6*	Unconventional myosin-VI	1.75	0.0009482
CISY_DANRE	*cs*	Citrate synthase	1.73	0.0008091
KPYM_CHICK	*PKM*	Pyruvate kinase PKM	1.71	0.0122511
UCRI_MOUSE	*Uqcrfs1*	Cytochrome b-c1 complex subunit Rieske	1.70	0.0031496
ACADM_RAT	*Acadm*	Medium-chain specific acyl-CoA dehydrogenase	1.70	0.0438522
SYPH_MOUSE	*Syp*	Synaptophysin	1.70	0.0041077
AT5F1_PONAB	*ATP5F1*	ATP synthase F(0) complex subunit B1	1.69	0.0064455
ADT3_BOVIN	*SLC25A6*	ADP/ATP translocase 3	1.69	0.0150770
NLS1B_DANRE	*mfsd2ab*	Sodium-dependent lysophosphatidylcholine symporter 1-B	1.68	0.0272424
ECHA_RAT	*Hadha*	Trifunctional enzyme subunit alpha	1.67	0.0027369
ADT2_BOVIN	*SLC25A5*	ADP/ATP translocase 2	1.66	0.0289562
ACON_BOVIN	*ACO2*	Aconitate hydratase	1.66	0.0006630
KCNV1_BOVIN	*KCNV1*	Potassium voltage-gated channel subfamily V member 1	1.65	0.0236049
PGAM2_HUMAN	*PGAM2*	Phosphoglycerate mutase 2	1.64	0.0458743
ECHB_MACFA	*HADHB*	Trifunctional enzyme subunit beta	1.64	0.0153694
NDUAA_HUMAN	*NDUFA10*	NADH dehydrogenase [ubiquinone] 1 alpha subcomplex subunit 10	1.64	0.0027091
ADT1_BOVIN	*SLC25A4*	ADP/ATP translocase 1	1.64	0.0373976
NDUS4_HUMAN	*NDUFS4*	NADH dehydrogenase [ubiquinone] iron-sulfur protein 4	1.61	1.724 × 10^−5^
MYOZ2_PONAB	*MYOZ2*	Myozenin-2	1.60	0.0020216

**Note**: *N/A*: Not applicable; FC: fold changes.

**Table 2:** The up-regulated DEPs in yellow skin (Y) compared to black skin (B).

Uniprot_ID	Gene	Description	FC(Y/B)	*p* Value
FXR2_HUMAN	*FXR2*	Fragile X mental retardation syndrome-related protein 2	5.18	0.0018055
MYPC2_MOUSE	*Mybpc2*	Myosin-binding protein C	3.99	0.0101018
TPM1_LIZAU	*N/A*	Tropomyosin alpha-1 chain	3.21	0.0026475
PRV7_DANRE	*pvalb7*	Parvalbumin-7	2.86	0.0203993
TPM1_XENLA	*tpm1*	Tropomyosin alpha-1 chain	2.57	0.0042248
CP110_HUMAN	*CCP110*	Centriolar coiled-coil protein of 110 kDa	2.45	0.0203727
LEG12_HUMAN	*LGALS12*	Galectin-12	1.73	0.0051317
H11_HUMAN	*HIST1H1A*	Histone H1.1	1.68	0.0206282
COCA1_MOUSE	*Col12a1*	Collagen alpha-1(XII) chain	1.67	0.0363538
LEG4_BOVIN	*LGALS4*	Galectin-4	1.66	0.0101980
TITIN_MOUSE	*Ttn*	Titin	1.62	0.0323899
TITIN_HUMAN	*TTN*	Titin	1.62	0.0486235
RHOG_HUMAN	*RHOG*	Rho-related GTP-binding protein RhoG	1.59	0.0213023
COCA1_HUMAN	*COL12A1*	Collagen alpha-1(XII) chain	1.56	0.0135574
CAHZ_DANRE	*cahz*	Carbonic anhydrase	1.45	0.0387359
C2D1B_XENLA	*cc2d1b*	Coiled-coil and C2 domain-containing protein 1B	1.43	0.0146113
PLPP1_CAVPO	*PLPP1*	Phospholipid phosphatase 1	1.42	0.0265325
EIF1B_HUMAN	*EIF1B*	Eukaryotic translation initiation factor 1b	1.42	0.0201610
BRE1A_HUMAN	*RNF20*	E3 ubiquitin-protein ligase BRE1A	1.40	0.0237980
HCE1_ORYLA	*hcea*	High choriolytic enzyme 1	1.35	0.0377692
SPTB2_MOUSE	*Sptbn1*	Spectrin beta chain	1.34	0.0234210
HBB1_DANRE	*ba1*	Hemoglobin subunit beta-1	1.34	0.0356035
B3AT_ONCMY	*slc4a1*	Band 3 anion exchange protein	1.33	0.0495074
DMD_PIG	*DMD*	Dystrophin	1.33	0.0089534
AQP3_MOUSE	*Aqp3*	Aquaporin-3	1.32	0.0099533
H3_DROME	*His3*	Histone H3.2	1.32	0.0314604
MCM7_XENTR	*mcm7*	DNA replication licensing factor mcm7	1.32	0.0348232
H2B12_XENLA	*N/A*	Histone H2B 1.2	1.31	0.0439092
NLRC3_HUMAN	*NLRC3*	Protein NLRC3	1.31	0.0217364
GOLP3_MOUSE	*Golph3*	Golgi phosphoprotein 3	1.30	0.0116354
TSP1_XENLA	*thbs1*	Thrombospondin-1	1.30	0.0151795
PSA2_CARAU	*psma2*	Proteasome subunit alpha type-2	1.29	0.0222874
FKBP5_HUMAN	*FKBP5*	Peptidyl-prolyl cis-trans isomerase FKBP5	1.29	0.0071276
FLNC_RAT	*Flnc*	Filamin-C	1.29	0.0458236
RALB_MACFA	*RALB*	Ras-related protein Ral-B	1.26	0.0165614
CP3AA_MESAU	*CYP3A10*	Lithocholate 6-beta-hydroxylase	1.26	0.0305838
CD63_MOUSE	*Cd63*	CD63 antigen	1.25	0.0002011
ZYX_CHICK	*ZYX*	Zyxin	1.24	0.0092652
CATL_SARPE	*N/A*	Cathepsin L	1.24	0.0087340
PTX3_MOUSE	*Ptx3*	Pentraxin-related protein PTX3	1.23	0.0302834
CATZ_RAT	*Ctsz*	Cathepsin Z	1.23	0.0072279
DYHC1_MOUSE	*Dync1h1*	Cytoplasmic dynein 1 heavy chain 1	1.23	0.0327325
AR6P6_BOVIN	*ARL6IP6*	ADP-ribosylation factor-like protein 6-interacting protein 6	1.23	0.0445232
TPM3_MOUSE	*Tpm3*	Tropomyosin alpha-3 chain	1.23	0.0293024
TBD2B_MOUSE	*Tbc1d2b*	TBC1 domain family member 2B	1.22	0.0450312
NEBU_HUMAN	*NEB*	Nebulin	1.22	0.0288621
LSM3_MOUSE	*Lsm3*	U6 snRNA-associated Sm-like protein LSm3	1.21	0.0099663
TXB1B_DANRE	*tax1bp1b*	Tax1-binding protein 1 homolog B	1.21	0.0473674
HEPH_MOUSE	*Heph*	Hephaestin	1.21	0.0252919
CAD26_HUMAN	*CDH26*	Cadherin-like protein 26	1.21	0.0214089
NIBL1_HUMAN	*FAM129B*	Niban-like protein 1	1.21	0.0188033
CNN1_MUSPF	*CNN1*	Calponin-1	1.21	0.0017860

**Table 3:** The DEPs up-regulated in W compared to Y (FC ≥ 1.6).

Uniprot_ID	Gene	Description	FC(W/Y)	*p* Value
HIBCH_DANRE	*hibch*	3-hydroxyisobutyryl-CoA hydrolase	4.52	0.0106552
RD23A_MOUSE	*Rad23a*	UV excision repair protein RAD23 homolog A	3.47	0.0130752
MREG_DANRE	*mreg*	Melanoregulin	3.20	3.6735 × 10^−5^
LAMB1_HUMAN	*LAMB1*	Laminin subunit beta-1	3.18	0.0192734
FBXL7_MOUSE	*Fbxl7*	F-box/LRR-repeat protein 7	2.48	0.0064965
MX2_ONCMY	*mx2*	Interferon-induced GTP-binding protein Mx2	2.43	0.0299254
K1C1_CARAU	*N/A*	Keratin	2.36	0.0008656
ST2B1_MOUSE	*Sult2b1*	Sulfotransferase family cytosolic 2B member 1	2.27	0.0020935
TBCB_BOVIN	*TBCB*	Tubulin-folding cofactor B	2.26	0.0141326
CMYA5_HUMAN	*CMYA5*	Cardiomyopathy-associated protein 5	2.17	0.0476189
NFL_COTJA	*NEFL*	Neurofilament light polypeptide	2.14	0.0275144
ETFA_HUMAN	*ETFA*	Electron transfer flavoprotein subunit alpha	1.97	0.0429857
MYPC3_CHICK	*MYBPC3*	Myosin-binding protein C	1.92	0.0243995
RDH12_HUMAN	*RDH12*	Retinol dehydrogenase 12	1.92	0.0340642
CWC22_DANRE	*cwc22*	Pre-mRNA-splicing factor CWC22 homolog	1.89	0.0230742
AT2A1_MAKNI	*atp2a1*	Sarcoplasmic/endoplasmic reticulum calcium ATPase 1	1.85	0.0471695
PLEC_MOUSE	*Plec*	Plectin	1.84	0.0021985
GSE1_DANRE	*gse1*	Genetic suppressor element 1	1.83	0.0457959
PLEC_HUMAN	*PLEC*	Plectin	1.81	0.0268665
OPA1_DANRE	*opa1*	Dynamin-like 120 kDa protein	1.81	0.0430209
LAMA4_HUMAN	*LAMA4*	Laminin subunit alpha-4	1.81	0.0229941
DCXR_MESAU	*DCXR*	L-xylulose reductase	1.81	0.0028180
A2MP_MOUSE	*A2m*	Alpha-2-macroglobulin-P	1.80	0.0145305
MF2NB_DANRE	*borcs8*	Protein MEF2BNB	1.80	0.0487061
SYK_HUMAN	*KARS*	Lysine—tRNA ligase	1.80	0.0010683
SPAG7_DANRE	*spag7*	Sperm-associated antigen 7 homolog	1.76	0.0268045
COX42_THUOB	*N/A*	Cytochrome c oxidase subunit 4 isoform 2	1.76	0.0198968
AT2A1_RABIT	*ATP2A1*	Sarcoplasmic/endoplasmic reticulum calcium ATPase 1	1.75	0.0471735
NUP98_RAT	*Nup98*	Nuclear pore complex protein Nup98-Nup96	1.75	0.0376341
ALKB5_DANRE	*alkbh5*	RNA demethylase ALKBH5	1.73	0.0135027
CLIC2_HUMAN	*CLIC2*	Chloride intracellular channel protein 2	1.72	0.0347927
TYRP1_CARAU	*tyrp1*	5,6-dihydroxyindole-2-carboxylic acid oxidase5	1.72	0.0002676
RRBP1_HUMAN	*RRBP1*	Ribosome-binding protein 1	1.71	0.0366481
SRSF5_HUMAN	*SRSF5*	Serine/arginine-rich splicing factor 5	1.70	0.0002839
COCA1_CHICK	*COL12A1*	Collagen alpha-1(XII) chain	1.70	0.0262727
AGRB2_MOUSE	*Adgrb2*	Adhesion G protein-coupled receptor B2	1.70	0.0044674
LPIN1_HUMAN	*LPIN1*	Phosphatidate phosphatase LPIN1	1.69	0.0197062
PLIN5_RAT	*Plin5*	Perilipin-5	1.69	0.0165470
CALD1_MELGA	*CALD1*	Caldesmon	1.67	0.0016593
PMEL_CHICK	*PMEL*	Melanocyte protein PMEL	1.67	0.0316520
RM43_BOVIN	*MRPL43*	39S ribosomal protein L43	1.67	0.0311367
AIP_RAT	*Aip*	AH receptor-interacting protein	1.66	0.0455057
MRP_BOVIN	*MARCKSL1*	MARCKS-related protein	1.65	0.0044254
PSMF1_PONAB	*PSMF1*	Proteasome inhibitor PI31 subunit	1.65	0.0351891
PTX3_HUMAN	*PTX3*	Pentraxin-related protein PTX3	1.64	0.0357322
HSP7E_DANRE	*hspa14*	Heat shock 70 kDa protein 14	1.64	0.0100857
K2C8_HUMAN	*KRT8*	Keratin	1.64	0.0073882
TPD54_HUMAN	*TPD52L2*	Tumor protein D54	1.63	0.0281041
PGS2_BOVIN	*DCN*	Decorin	1.63	0.0046897
TRI11_RAT	*Trim11*	E3 ubiquitin-protein ligase TRIM11	1.63	0.0092775
RPF2_BOVIN	*RPF2*	Ribosome production factor 2 homolog	1.63	0.0380427
ECHD1_DANRE	*echdc1*	Ethylmalonyl-CoA decarboxylase	1.63	0.0064541
SNAB_HUMAN	*NAPB*	Beta-soluble NSF attachment protein	1.62	0.0255251
PKP3_MOUSE	*Pkp3*	Plakophilin-3	1.61	0.0044704
SSF1_MOUSE	*Ppan*	Suppressor of SWI4 1 homolog	1.61	0.0288496
ADDG_HUMAN	*ADD3*	Gamma-adducin	1.61	0.0010349
PBX1_HUMAN	*PBX1*	Pre-B-cell leukemia transcription factor 1	1.60	0.0001923
RTN1_PANTR	*RTN1*	Reticulon-1	1.60	0.0389269

**Note**: *N/A*: Not applicable.

**Table 4:** The DEPs up-regulated in Y compared to W (FC ≥ 1.5).

Uniprot_ID	Gene	Description	FC(Y/W)	*p* Value (W/Y)
MYOM2_HUMAN	*MYOM2*	Myomesin-2	4.87	0.0034066
DDR2_MOUSE	*Ddr2*	Discoidin domain-containing receptor 2	4.68	0.0283762
TNNC2_ANGAN	*N/A*	Troponin C	4.50	0.0072123
MYOZ1_HUMAN	*MYOZ1*	Myozenin-1	4.01	0.0065529
MYSS_CYPCA	*N/A*	Myosin heavy chain	3.53	0.0017226
PRV2_DANRE	*pvalb2*	Parvalbumin-2	3.34	0.0176125
MYOZ1_BOVIN	*MYOZ1*	Myozenin-1	3.25	0.0011707
ACTN3_BOVIN	*ACTN3*	Alpha-actinin-3	3.09	0.0014259
PDLI7_BOVIN	*PDLIM7*	PDZ and LIM domain protein 7	3.05	0.0062208
SAHHB_XENLA	*ahcy-b*	Adenosylhomocysteinase B	3.01	0.0097743
TITIN_HUMAN	*TTN*	Titin	2.97	0.0040666
DYST_HUMAN	*DST*	Dystonin	2.90	0.0213198
MYBPH_CHICK	*MYBPH*	Myosin-binding protein H	2.83	0.0015750
CASR_BOVIN	*CASR*	Extracellular calcium-sensing receptor	2.81	0.0064296
NRG_DROME	*Nrg*	Neuroglian	2.75	0.0083956
MTUS2_HUMAN	*MTUS2*	Microtubule-associated tumor suppressor candidate 2	2.74	0.0214764
MYPC2_MOUSE	*Mybpc2*	Myosin-binding protein C	2.72	0.0059417
AMPD1_HUMAN	*AMPD1*	AMP deaminase 1	2.61	0.0074214
MAP7_CHICK	*MAP7*	Ensconsin	2.61	0.0064867
TNNT3_COTJA	*TNNT3*	Troponin T	2.60	8.4696 × 10^−5^
GIMA5_HUMAN	*GIMAP5*	GTPase IMAP family member 5	2.29	0.0243491
AOXC_MOUSE	*Aox3*	Aldehyde oxidase 3	2.22	0.0150366
K1C14_MOUSE	*Krt14*	Keratin	2.17	0.0462584
TNNI2_RABIT	*TNNI2*	Troponin I	2.16	0.0005866
KPYM_CHICK	*PKM*	Pyruvate kinase PKM	2.16	0.0370822
MPSF_CHICK	*N/A*	M-protein	2.13	0.0065892
KCRM_CANFA	*CKM*	Creatine kinase M-type	2.11	0.0154615
LDB3_HUMAN	*LDB3*	LIM domain-binding protein 3	2.10	0.0389130
FUCO_HUMAN	*FUCA1*	Tissue alpha-L-fucosidase	2.06	0.0105965
MYO1F_HUMAN	*MYO1F*	Unconventional myosin-If	2.04	0.0272882
KPCD_HUMAN	*PRKCD*	Protein kinase C delta type	2.01	0.0146456
FLNC_RAT	*Flnc*	Filamin-C	2.01	0.0345344
TPM3_HUMAN	*TPM3*	Tropomyosin alpha-3 chain	1.99	0.0062754
RNT2_DANRE	*rnaset2*	Ribonuclease T2	1.99	0.0007026
CHP3_XENLA	*tesc*	Calcineurin B homologous protein 3	1.98	0.0098880
VTNC_RABIT	*VTN*	Vitronectin	1.97	0.0419940
ACTSA_TAKRU	*acta1a*	Actin	1.94	0.0035608
FUCO_MOUSE	*Fuca1*	Tissue alpha-L-fucosidase	1.91	0.0197286
MYH4_HUMAN	*MYH4*	Myosin-4	1.86	0.0243060
IF44L_MOUSE	*Ifi44l*	Interferon-induced protein 44-like	1.86	0.0264745
BAX_BOVIN	*BAX*	Apoptosis regulator BAX	1.84	0.0027166
NEBU_HUMAN	*NEB*	Nebulin	1.84	0.0041143
AKA12_HUMAN	*AKAP12*	A-kinase anchor protein 12	1.82	0.0224758
EF1A1_HORSE	*EEF1A1*	Elongation factor 1-alpha 1	1.81	0.0125514
K1C13_ONCMY	*krt13*	Keratin	1.80	0.0162506
FUCM_DANRE	*fuom*	Fucose mutarotase	1.77	0.0014529
TIMP3_XENLA	*timp3*	Metalloproteinase inhibitor 3	1.74	0.0209090
CNIH4_BOVIN	*CNIH4*	Protein cornichon homolog 4	1.72	0.0420714
PDLI7_MOUSE	*Pdlim7*	PDZ and LIM domain protein 7	1.70	0.0173236
CATB_CHICK	*CTSB*	Cathepsin B	1.69	0.0004048
OBSCN_MOUSE	*Obscn*	Obscurin	1.68	0.0015550
MYOZ2_PONAB	*MYOZ2*	Myozenin-2	1.66	0.0485948
SAP_CHICK	*PSAP*	Prosaposin	1.66	0.0389310
PUA1A_SALSA	*adssl1a*	Adenylosuccinate synthetase isozyme 1 A	1.65	0.0115606
CFAB_BOVIN	*CFB*	Complement factor B	1.64	0.0096244
LEG12_HUMAN	*LGALS12*	Galectin-12	1.63	0.0231777
WDR47_MOUSE	*Wdr47*	WD repeat-containing protein 47	1.62	0.0030872
ALDOA_MOUSE	*Aldoa*	Fructose-bisphosphate aldolase A	1.62	0.0033610
CFAB_MOUSE	*Cfb*	Complement factor B	1.60	0.0027860
G3P_CHICK	*GAPDH*	Glyceraldehyde-3-phosphate dehydrogenase	1.58	0.0436886
DMD_CHICK	*DMD*	Dystrophin	1.56	0.0007040
PPGB_MOUSE	*Ctsa*	Lysosomal protective protein	1.55	0.0207043
DPP2_MOUSE	*Dpp7*	Dipeptidyl peptidase 2	1.54	0.0001501
TITIN_MOUSE	*Ttn*	Titin	1.54	0.0002004
NUPR2_HUMAN	*NUPR2*	Nuclear protein 2	1.53	0.0135287
LGMN_BOVIN	*LGMN*	Legumain	1.53	0.0159403
BGLR_MOUSE	*Gusb*	Beta-glucuronidase	1.53	0.0195223
CATH_PIG	*CTSH*	Pro-cathepsin H	1.52	0.0364061
ING5_HUMAN	*ING5*	Inhibitor of growth protein 5	1.52	0.0011046
ALDOA_SALSA	*N/A*	Fructose-bisphosphate aldolase A	1.51	0.0039936
CAPZB_CHICK	*CAPZB*	F-actin-capping protein subunit beta isoforms 1 and 2	1.51	0.0220029

**Note**: *N/A*: Not applicable.

## Supplementary Materials

The following are available online at https://www.mdpi.com/2075-1729/10/10/226/s1, Table S1: Raw data analysis of the proteomic data among yellow skin in Jinbian carp (Y), black skin in Jinbian carp (B), and black skin in wild common carp (W).
